# Resonance-State Temperature Compensation Method for Ultrasonic Resonance Wind Speed and Direction Sensors

**DOI:** 10.3390/s24227217

**Published:** 2024-11-12

**Authors:** Xiangbao Zeng, Yupeng Yuan, Zhuoya Jiang, Lu Wang, Shuai Long, Hua Yu

**Affiliations:** 1 College of Optoelectronic Engineering, Chongqing University, Chongqing 400040, China; xiangbaozeng@sina.com (X.Z.); zhuoya_jiang@163.com (Z.J.); 2 The 26th Institute of China Electronics Technology Group Corporation, Chongqing 400060, China; yuanyp@cetccq.com.cn (Y.Y.); wanglu@sina.com (L.W.); longshuai@sina.com (S.L.)

**Keywords:** wind speed and direction sensor, resonance-state temperature compensation, adaptive resonance-state tracking, wind speed measurement

## Abstract

To achieve high-precision wind speed and direction measurements in complex environments, a resonance-state temperature compensation method is proposed based on an ultrasonic resonance principle. This method effectively addresses the issue of sound velocity compensation errors caused by the temperature difference between the internal and external environments when using an internal temperature sensor for temperature compensation. By utilizing an adaptive resonance-state tracking model, the resonance frequency shift issues under varying conditions such as altitude, pressure, and temperature are mitigated. This approach ensures that the resonance frequency is strongly correlated with temperature, enabling temperature compensation through resonance frequency alone, without the need for a temperature sensor. The experimental results indicate that the resonance frequency variation rate with temperature for the resonance-state temperature-compensated ultrasonic resonance wind speed and direction sensor is approximately 0.08 kHz/°C. The wind speed measurement accuracy is ±0.3 m/s (≤15 m/s)/±2.3% (15 m/s~50 m/s), which is superior to the measurement accuracy of traditional ultrasonic wind speed and direction sensors (±0.5 m/s (≤15 m/s)/±4% (15 m/s~50 m/s)). The consistency of wind speed measurement is ≤±0.3%, representing an improvement of approximately 3% compared to ultrasonic resonance wind speed and direction sensors without resonance-state temperature compensation.

## 1. Introduction

Wind is one of the most common natural phenomena in nature, caused by the movement of air. Wind speed, defined as the horizontal distance that air moves relative to a fixed point on Earth over a specific time, is measured in meters per second (m/s). Wind direction is described as the angle from a fixed reference direction, ranging from 0° to 360°, indicating the origin of the airflow. The precise measurement of wind speed and direction is of significant importance in various societal production and daily life activities. In agricultural production, wind facilitates the dispersal of pollen, enabling the pollination and reproduction of crops. However, hurricanes can cause significant destruction to agricultural activities. Therefore, accurately measuring wind speed and direction in agricultural environments is crucial for ensuring the safety of production processes [1]. In the context of transportation, the safe operation of vehicles such as ships and high-speed trains requires monitoring wind speed and direction during their journeys to prevent interference from crosswinds. In meteorological forecasting, monitoring wind speed and direction serves as an important reference for predicting destructive weather phenomena like typhoons and hurricanes. This information is essential for forecasting weather trends and safeguarding public life and property [2].

Wind speed and direction sensors are widely used in wind power generation, aviation, and meteorology. Currently, wind speed and direction sensors primarily include mechanical, ultrasonic, hot-wire, laser, and micro-electro-mechanical system (MEMS)-based types. Ultrasonic wind speed and direction sensors, due to their high sensitivity, precision, and the advantage of having no startup wind speed, have gradually replaced traditional mechanical sensors as the mainstream technology. However, traditional ultrasonic wind speed and direction sensors face issues such as large size, center frequency drift over extended periods of operation leading to wind speed measurement errors, inaccurate temperature compensation, and poor environmental adaptability.

To address the limitations of traditional ultrasonic transit/reflective wind speed and direction sensors, an ultrasonic resonance wind speed and direction sensor was designed and developed. This sensor is compact and drift-free over long periods and has strong environmental adaptability. However, due to its fully enclosed design, there is a temperature difference between the actual operating environment and the internal temperature measured by the sensor, leading to inaccurate temperature compensation and, consequently, errors in wind speed and direction measurements. Recently, Shan ZB proposed methods to effectively reduce the impact of temperature, thereby improving the accuracy of wind speed measurements [3]; there are two methods to address the temperature compensation issue. The first method involves performing temperature compensation on the collected time signals by estimating the temperature based on the propagation time of the ultrasonic wave in the air. However, this method is ineffective due to interface effects and time sampling issues. The second method uses a Wheatstone bridge in combination with a thermistor, i.e., a temperature sensor, which also suffers from internal and external temperature differences, leading to inaccurate temperature compensation [4,5].

The ultrasonic resonance wind speed and direction sensor utilizes the principle of ultrasonic resonance for wind speed and direction measurement; its resonance frequency changes with variations in temperature, resonance cavity height, and pressure. However, since the height of the ultrasonic resonance wind speed and direction sensor remains largely unchanged after assembly, and the working environment is a typical natural environment with relatively stable pressure, the primary factor affecting the resonance frequency is the ambient temperature. Therefore, a resonance-state temperature compensation method is proposed to address the issue of inaccurate temperature compensation. A comparison of various temperature compensation methods is shown in Table 1.

## 2. Principle of Wind Speed and Direction Measurement Using an Ultrasonic Resonance Wind Sensor

**Figure 1:**
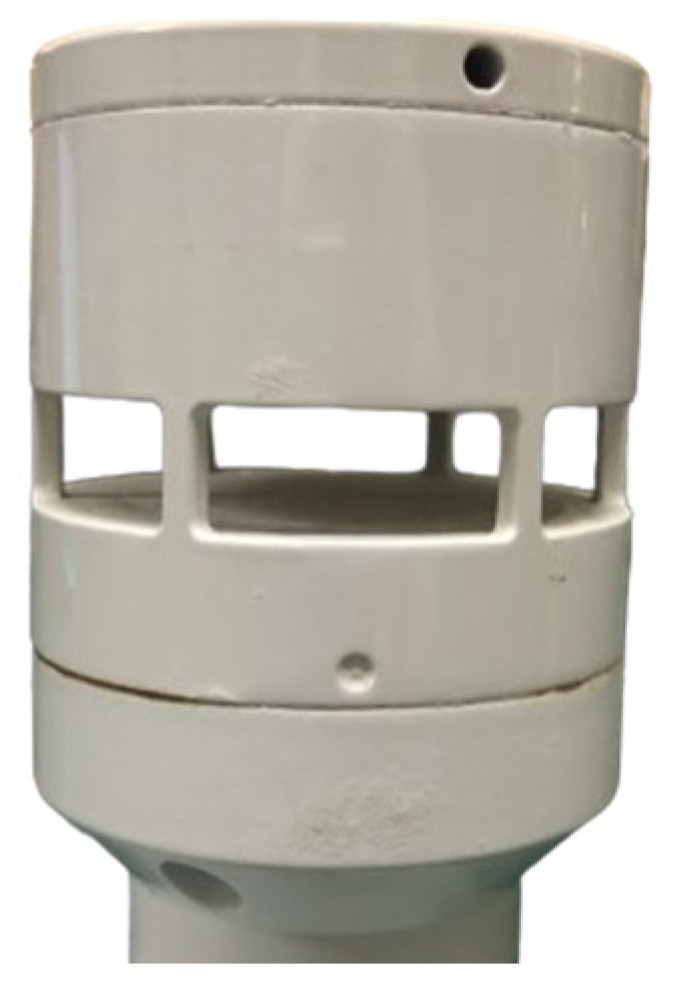
Ultrasonic resonance wind speed and direction sensor.

The ultrasonic resonance wind speed and direction sensor operates on the principle of ultrasonic resonance, characterized by its compact size, high precision, and long-term stability without drift. The physical device is shown in Figure 1.

**Figure 2:**
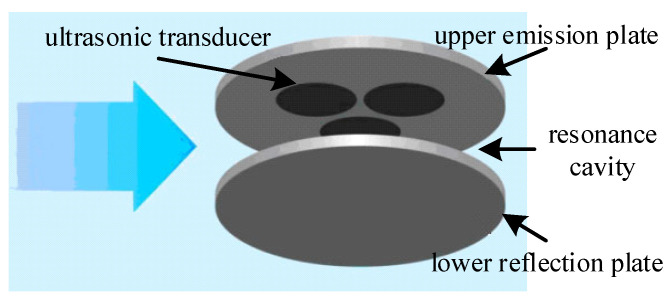
Schematic of wind measurement using the ultrasonic resonance wind sensor.

The operational principle of the ultrasonic resonance wind speed and direction sensor is illustrated in Figure 2. It primarily utilizes the resonance of ultrasonic waves within a small chamber to measure wind speed and direction. The sensor mainly comprises an upper emission plate, a lower reflection plate, a resonance cavity, and three cylindrical piezoelectric ultrasonic transducers fixed on the upper emission plate. Horizontally, the structure is unobstructed and without boundaries, allowing air to flow freely. Vertically, the structure is constrained, preventing free air movement; hence, the vertical wind speed can be neglected, allowing for two-dimensional wind speed and direction measurement.

**Figure 3:**
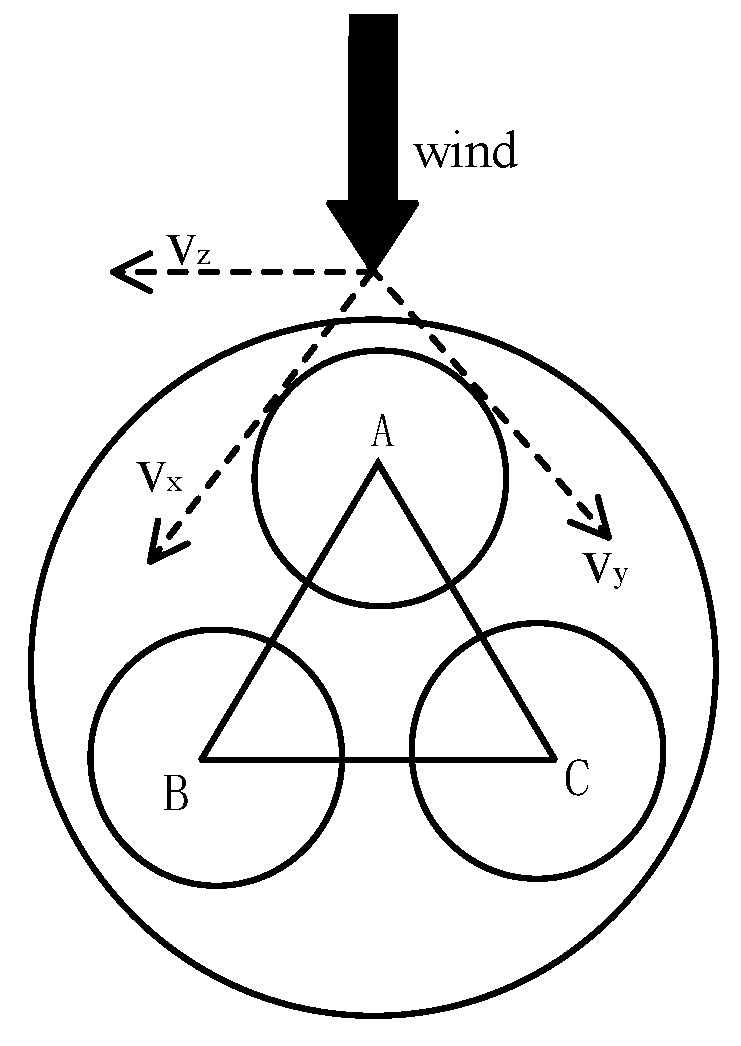
Wind measurement calculation model.

To achieve wind speed and direction measurement, an ultrasonic array arranged in an equilateral triangle is required, as shown in the measurement model in Figure 3. When wind passes through the equilateral triangular ultrasonic array, the ultrasonic waves emitted by the array experience corresponding time shifts or phase shifts due to the wind’s influence [6]. By calculating the time shifts of the three sides of the triangle, the wind speed and direction can be determined through computation.

The ultrasonic wind speed and direction measurement model is a self-constructed model developed based on the fundamental measurement principle of the Time-Of-Flight Diffraction [7]. The formula represents the derivation results of the new model.

In Figure 3 *A*, *B*, and *C* indicate the central points of the ultrasonic transducers. Taking point *A* as the vertex, when wind blows, it creates wind speed components along the three directions of the equilateral triangle. These components cause different arrival times in each direction. Here, the time difference method is employed, involving mutual transmission and reception between the directions. The times for each direction component are as follows: *A* emits, *B* receives, and the time is *T_AB_*; *B* emits, *A* receives, and the time is *T_BA_*; *A* emits, *C* receives, and the time is *T_CA_*; *B* emits, *C* receives, and the time is *T_BC_*; and *C* emits, *B* receives, and the time is *T_CB_*. Since the distance *L* is fixed, we have the following [8,9]:

(1) $$T_{AB}+T_{\mathrm{LinkAB}}+T_{\mathrm{DelayAB}}=\frac{L}{c-v_{x}},$$

$T_{\mathrm{LinkAB}}$ is the time it takes for the signal to reach the transducer from the transmitting circuit. $T_{\mathrm{DelayAB}}$ is the time it takes for the signal to reach the MCU from the receiving circuit. The speed of ultrasonic waves can be expressed as follows [7]:

(2) $$c=\sqrt{\frac{\gamma RT}{M}},$$

where γ represents the ratio of specific heats at constant pressure $C_{P}$ and constant volume $C_{V}$, *R* represents the universal gas constant, *T* represents the absolute temperature, and *M* represents the molecular weight of the gas. From Equations (1) and (2), we can calculate the following:

(3) $$T_{AB}=\frac{L}{\sqrt{\frac{\gamma RT}{M}}-v_{x}}-T_{\mathrm{LinkAB}}-T_{\mathrm{DelayAB}},$$

Similarly, we obtain the following:

(4) $$T_{BA}=\frac{L}{\sqrt{\frac{\gamma RT}{M}}+v_{x}}-T_{\mathrm{LinkAB}}-T_{\mathrm{DelayAB}},$$

Based on the actual design, the circuit ensures that $T_{\mathrm{LinkAB}}=T_{\mathrm{LinkAB}}$ and $T_{\mathrm{DelayAB}}=T_{\mathrm{DelayAB}}$, and by subtracting the two, intermediate influences are eliminated. From Equations (3) and (4), we calculate the following:

(5) $$△t_{x}=T_{AB}-T_{BA}=\frac{L}{\sqrt{\frac{\gamma RT}{M}}-v_{x}}-\frac{L}{\sqrt{\frac{\gamma RT}{M}}+v_{x}},$$

Similarly, we obtain the following:

(6) $$△t_{y}=T_{AC}-T_{CA}=\frac{L}{\sqrt{\frac{\gamma RT}{M}}-v_{y}}-\frac{L}{\sqrt{\frac{\gamma RT}{M}}+v_{y}},$$

(7) $$△t_{z}=T_{BC}-T_{CB}=\frac{L}{\sqrt{\frac{\gamma RT}{M}}-v_{Z}}-\frac{L}{\sqrt{\frac{\gamma RT}{M}}+v_{Z}},$$

By using a timer, the values of $△t_{x}$, $△t_{y}$, and $△t_{z}$ can be accurately obtained. The acoustic path length *L* is a fixed value, and the values of γ, R, and M are also constants, while *T* is the variable representing absolute temperature. Therefore, Equations (5)–(7) can be used to calculate the following:

(8) $$v_{x}=\sqrt{\frac{\gamma RT}{M}+\left(\frac{L^{2}}{△{t_{x}}^{2}}\right)}-\frac{L}{△t_{x}},$$

(9) $$v_{y}=\sqrt{\frac{\gamma RT}{M}+\left(\frac{L^{2}}{△{t_{y}}^{2}}\right)}-\frac{L}{△t_{y}},$$

(10) $$v_{z}=\sqrt{\frac{\gamma RT}{M}+\left(\frac{L^{2}}{△{t_{z}}^{2}}\right)}-\frac{L}{△t_{z}},$$

Based on trigonometric relationships, Equations (8) and (9) can be used to derive the wind speed:

(11) $$v=\sqrt{{\left(\sqrt{\frac{\gamma RT}{M}+\left(\frac{L^{2}}{△{t_{x}}^{2}}\right)}-\sqrt{\frac{\gamma RT}{M}+\left(\frac{L^{2}}{△{t_{y}}^{2}}\right)}+\frac{L}{△t_{y}}-\frac{L}{△t_{x}}\right)}^{2}+\frac{{\left(\sqrt{\frac{\gamma RT}{M}+\left(\frac{L^{2}}{△{t_{x}}^{2}}\right)}-\frac{L}{△t_{x}}+\sqrt{\frac{\gamma RT}{M}+\left(\frac{L^{2}}{△{t_{y}}^{2}}\right)}-\frac{L}{△t_{y}}\right)}^{2}}{3}},$$

Using Equations (10) and (11), the wind direction φ can be calculated as follows:

(12) $$\varphi =arc\sin\left(\frac{\sqrt{\frac{\gamma RT}{M}+\left(\frac{L^{2}}{△{t_{z}}^{2}}\right)}-\frac{L}{△t_{z}}}{\sqrt{{\left(\sqrt{\frac{\gamma RT}{M}+\left(\frac{L^{2}}{△{t_{x}}^{2}}\right)}-\sqrt{\frac{\gamma RT}{M}+\left(\frac{L^{2}}{△{t_{y}}^{2}}\right)}+\frac{L}{△t_{y}}-\frac{L}{△t_{x}}\right)}^{2}+\frac{{\left(\sqrt{\frac{\gamma RT}{M}+\left(\frac{L^{2}}{△{t_{x}}^{2}}\right)}-\frac{L}{△t_{x}}+\sqrt{\frac{\gamma RT}{M}+\left(\frac{L^{2}}{△{t_{y}}^{2}}\right)}-\frac{L}{△t_{y}}\right)}^{2}}{3}}}\right),$$

As evident from Equations (11) and (12), both wind speed and direction are correlated with temperature *T*. In practical applications, the accuracy of temperature directly affects the measurement precision of wind speed and direction. The calculations for wind speed and direction are derived from the components $v_{x}$, $v_{y}$, and $v_{z}$; for ease of calculation, subsequent discussions and calculations will use $v_{x}$ for analysis.

## 3. Analysis of Measurement Errors Induced by Temperature

**Figure 4:**
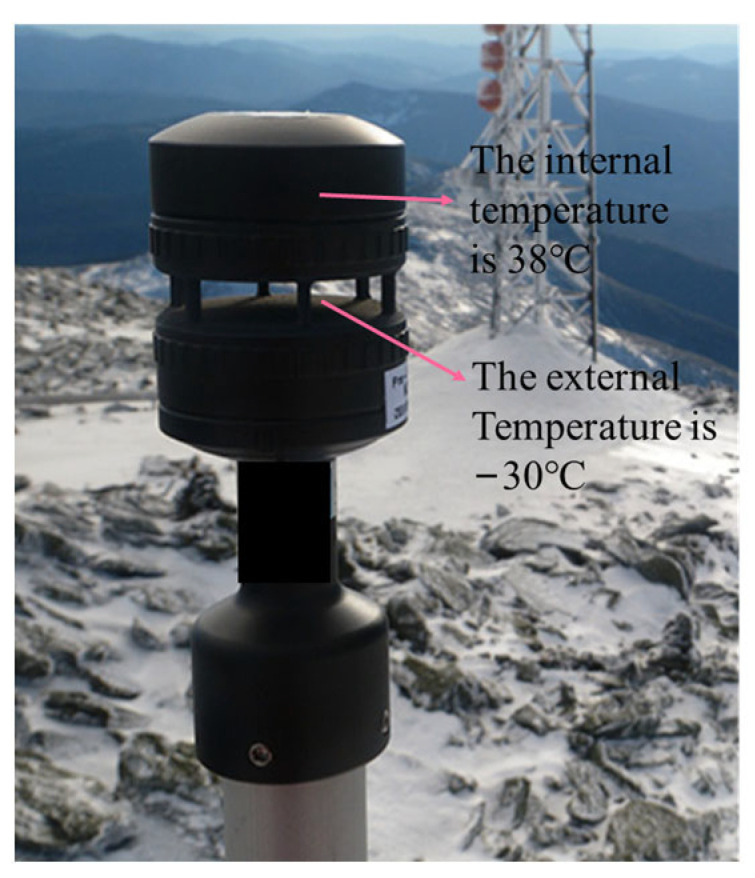
Actual operating temperature distribution of the sensor.

Traditional wind speed and direction sensors typically use built-in temperature sensors to measure and compensate for temperature. To meet the IP67 rating requirements, the temperature sensor is completely sealed inside the sensor housing. As a result, the temperature measured is that of the internal environment of the wind speed and direction sensor. However, since the ultrasonic wave operates in the external environment, there is often a significant temperature difference between the internal and external environments. This difference leads to inaccurate temperature compensation, which in turn causes deviations in the calculated wind speed and direction values, as illustrated in Figure 4.

As shown in Figure 4, when the ultrasonic resonance wind speed and direction sensor is in operation, the internal temperature is 38 °C, while the external temperature is −30 °C. The substantial temperature difference between the internal and external environments causes significant discrepancies in the sound velocity values used for calculation, resulting in considerable errors in the computed wind speed and direction [6,7,8,9].

For air, temperature is the primary influencing factor. This is according to the following empirical formula for the speed of sound:

(13) c=331.45+0.606×T,

where *T* is the temperature. Within the temperature range of −40 °C to 60 °C, the speed of sound ranges from 307.21 m/s to 367.81 m/s. This wide range of sound speed necessitates compensation in the calculations to minimize errors in wind speed and direction measurements.

From Equation (8), it is known that this value is fixed when the wind speed is determined. Through practical testing, it has been found that when the wind speed is 10 m/s, this value is approximately 315, and the overall expression can be written as follows:

(14) $$v_{x}=\sqrt{c^{2}+315^{2}}-315,$$

**Figure 5:**
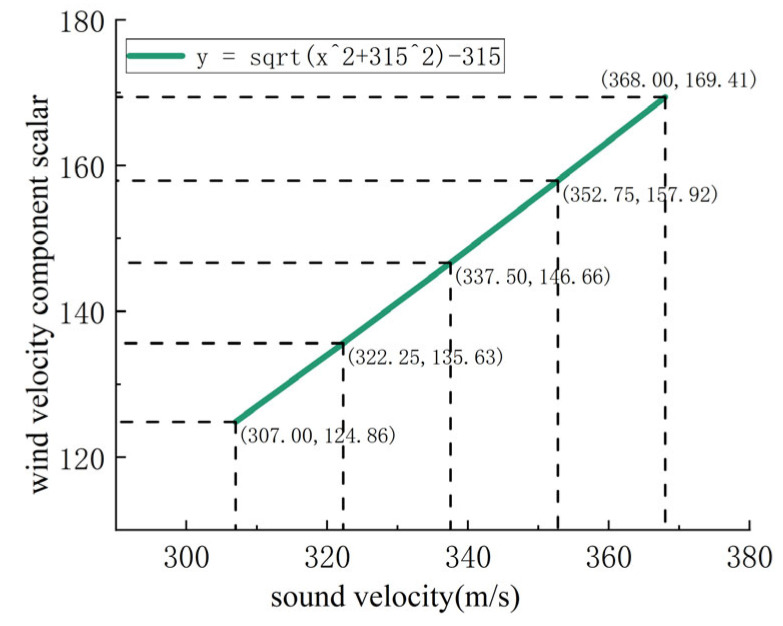
Speed of sound variation trend chart.

When the ultrasonic resonance wind speed and direction sensor is in operation, internal components generate a certain amount of heat, causing the internal temperature to be higher than the external temperature. At an external temperature of −40 °C, the internal temperature is approximately 10 °C, while at an external temperature of 10 °C, the internal temperature is around 60 °C. For the purpose of error analysis, 10 °C is used as a reference point, where the sound speed is approximately 337.5 m/s. Within the temperature range of −40 °C to 60 °C, the speed of sound varies from 307.21 m/s to 367.81 m/s. By substituting the speed of sound values into Equation (2), the corresponding trend chart is shown in Figure 5.

As depicted in Figure 5, when the speed of sound is 307 m/s, the wind speed component scalar is 124.86; when the speed of sound is 368 m/s, the wind speed component scalar is 169.41; under normal conditions, when the speed of sound is 337.5 m/s, the wind speed component scalar is 146.66. Calculations indicate that when the speed of sound is 368 m/s (corresponding to 60 °C), the error is approximately 15.5%, and when the speed of sound is 307 m/s (corresponding to −40 °C), the error is approximately 14.8%. Therefore, it can be concluded that using internal temperature compensation in ultrasonic resonance wind speed and direction sensors results in significant compensation errors, leading to calculation errors in wind speed and direction, making it challenging to achieve high-precision measurements of wind speed and direction.

## 4. Resonance-State Temperature Compensation Method

The ultrasonic resonance wind speed and direction sensor operates on the principle of ultrasonic resonance to measure wind speed and direction. Its resonance frequency changes with variations in temperature, resonance cavity height, and pressure. However, since the height of the ultrasonic resonance wind speed and direction sensor remains largely unchanged after assembly, and the working environment is a typical natural environment with relatively stable pressure, the primary factor affecting the resonance frequency is the ambient temperature. Therefore, the working temperature can be deduced from the ultrasonic resonance frequency during sensor operation, allowing for resonance-state temperature compensation.

In extreme or specific environmental conditions, the pressure can change significantly. In typical environments, the pressure is approximately 1 bar. The relationship between ultrasonic air propagation speed and pressure is complex. Simulation results for ultrasonic speed under various temperature and pressure conditions [10] are shown in Table 2; the remaining conditions are conventional.

As observed in Table 2, at the same temperature, the speed of sound varies by approximately 4 m/s when the pressure changes from 1 bar to 12 bar. Based on the principle of temperature compensation via ultrasonic resonance, the resonance point shifts by about 0.9%. Therefore, the impact of pressure changes on the practical application of resonance point temperature compensation is relatively minor.

### 4.1. Relationship Between Resonance State and Resonance Frequency

**Figure 6:**
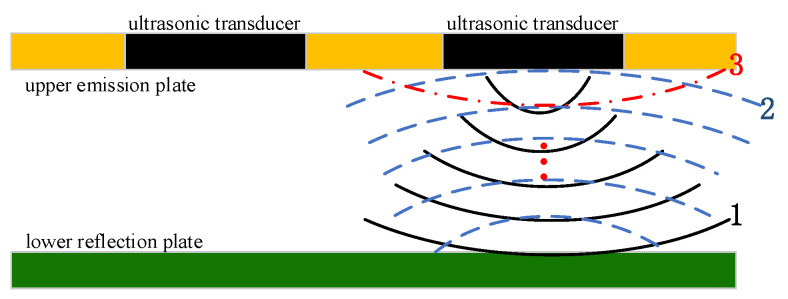
Schematic of ultrasonic plate resonance principle.

The ultrasonic resonance wind speed and direction sensor measures wind speed and direction based on the resonance of ultrasonic waves within flat plates [11]. It consists of two oppositely positioned plates that serve as the emission and reflection surfaces of the ultrasonic transducer. Three ultrasonic transducers are arranged on the emission surface in an equilateral triangle configuration, forming the basic ultrasonic plate resonance model. The ultrasonic transducers emit continuous waves at a specific frequency, and the emitted waves interfere with the reflected waves. Through repeated reflection and interference, stable standing waves are formed within the plates, causing ultrasonic resonance in the plates, essentially in the resonance of air columns within the enclosed space, as shown in Figure 6.

As seen in Figure 6, one transducer emits a continuous ultrasonic wave. The first emitted wave reaches the reflection surface (black line and number 1 in Figure 6) and, after reflection, forms the second ultrasonic signal (blue line and number 2 in Figure 6). The second signal reflects again, forming the third ultrasonic signal (red line and number 3 in Figure 6), and this process repeats cyclically. Due to the attenuation of the signal amplitude with each reflection, the ultrasonic signal eventually fully decays after several reflections [11]. Through repeated reflection, standing waves can form under specific conditions where the emitted wave and early reflected waves interfere constructively, thus satisfying the conditions for ultrasonic plate interference. If the first direct sound wave is represented as eikL (with amplitude set to 1), and the amplitude attenuation after each reflection is *δ (δ* < 1), the acoustic pressure on the receiving transducer can be expressed as follows [7]:

(15) $$Ae^{i\Phi }=\frac{e^{ikL}}{1-\delta e^{i\varphi }},$$

where *φ* = 2*kL* + *φr*, *k* = 2*πf/c*, *c* is the speed of sound, *L* is the distance, and *φr* represents the phase shift of acoustic pressure upon reflection between the emitting transducer and the plate. For air, *φr* = 0, and from Equation (15), we obtain the following [7]:

(16) $$A=\frac{1}{\sqrt{1-2\delta cos\varphi +\delta 2}},$$

From Equation (16), it is evident that resonance occurs when the amplitude reaches a maximum value. To achieve the maximum resonance amplitude, the resonance point can be adjusted by changing either *f* or *L*. In practical applications, *L* is a fixed distance, so resonance is achieved by varying the frequency *f*.

### 4.2. Adaptive Resonance-State Tracking Model

An ultrasonic resonance wind speed and direction sensor was designed based on the principle of ultrasonic resonance. However, during actual use, factors such as manufacturing and assembly processes can lead to inconsistent cavity heights. Additionally, the speed of sound and the wavelength of ultrasonic waves vary with temperature, causing wind speed measurement errors. To ensure stable resonance under all conditions and achieve temperature compensation during resonance, an adaptive resonance-state tracking model is proposed for the ultrasonic resonance wind speed and direction sensor.

**Figure 7:**
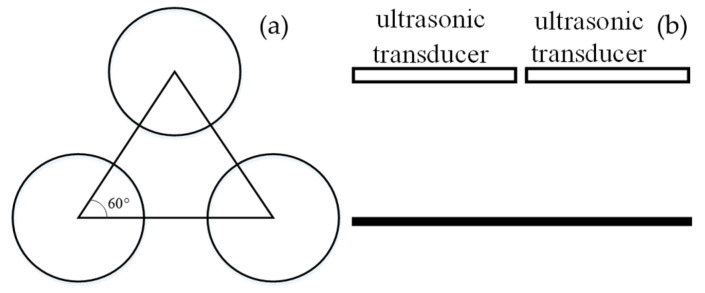
Schematic of ultrasonic transducer arrangement: (**a**) the top view; (**b**) the front view.

The sensor operates by utilizing the principle of acoustic interference to induce the resonance of the air columns within the cavity, achieving ultrasonic resonance. The sensor places three transducers in a single plane, forming an ultrasonic transducer array, as shown in Figure 7. Figure 7a shows the top view, and Figure 7b shows the front view.

The three ultrasonic transducers are arranged on the same plane, forming an equilateral triangle at the center, and together with the reflecting plate below, they create a cavity with a specific height. Ultrasonic signals propagate within the cavity, forming a fixed acoustic resonance beam [12,13].

It is assumed that the cavity height formed by the plates is *L*, and the ultrasonic wave emission frequency is *f*. This resonance formation method involves a single ultrasonic transducer emitting an ultrasonic signal at a specific frequency. The signal reflects back and forth between the two planes, and the reflected signal interferes with the emitted signal. When the wavelength of the emitted ultrasonic wave is an integer multiple of the half-wavelength of the cavity height, a stable resonance beam can form within the cavity.

The condition for ultrasonic resonance formation is that the cavity must satisfy the requirement of being an integer multiple of the half-wavelength of the emitted wave, expressed as follows:

(17) $$L=\frac{\lambda }{2}\times n,$$

The relationship between the wavelength, frequency, and speed of sound for the ultrasonic transducer is given by the following:

(18) $$\lambda =\frac{c}{f},$$

where the speed of sound *c* is a variable that changes with temperature. The formula for calculating *c* is given by the following: $c=\sqrt{KRT}$ where *K* is the ratio of specific heats at constant pressure and constant volume, *R* is the gas constant, and *T* is the absolute temperature. Ultimately, the beamforming condition can be expressed as follows:

(19) $$L=\frac{\sqrt{KRT}}{2f}\times n,$$

From Equation (19), we obtain the following:

(20) $$f=\frac{\sqrt{KRT}}{2L}\times n,$$

**Figure 8:**
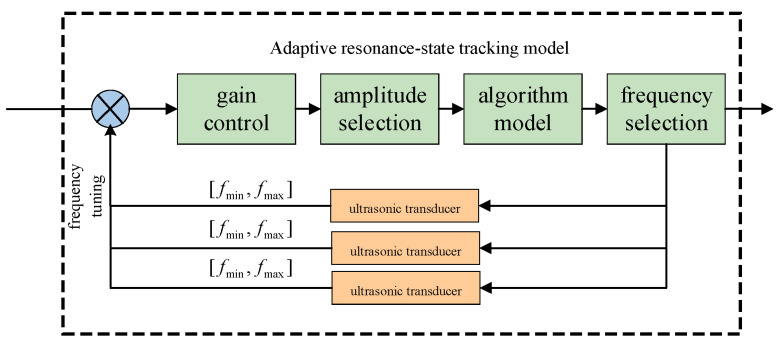
Block diagram of adaptive resonance-state tracking model.

In practice, *K*, *R*, *T*, and *L* are environmental variables. To adapt to these variables, the ultrasonic transducer performs a frequency scan. Due to the characteristics of ultrasonic resonance beamforming, the amplitude is maximized under the resonance state. By tracking the amplitude through frequency scanning, it is possible to track the resonance frequency under different conditions [14,15,16,17]. The block diagram of the adaptive beamforming principle is shown in Figure 8.

Through the adaptive ultrasonic resonance-state model, the frequency range of the ultrasonic transducer scan is set from *f*_min_ to *f*_max_. The amplitude model determines whether the resonance state is achieved. If it is, the current resonance frequency point is selected; if not, the range of *f*_min_ and *f*_max_ is adjusted until a suitable resonance frequency point is found.

**Figure 9:**
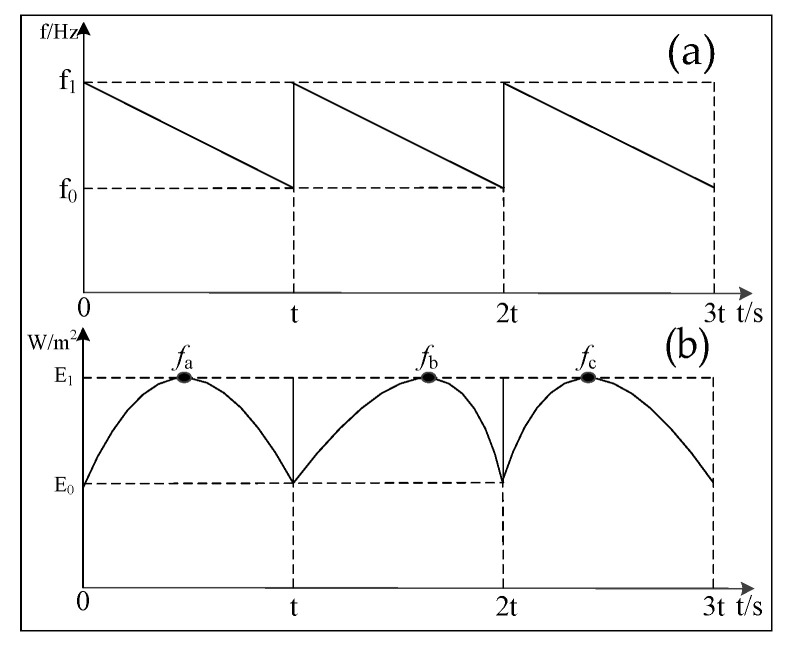
Adaptive resonance-state tracking scanning schematic: (**a**) the frequency scanning schematic; (**b**) the resonance frequency point position schematic.

The adaptive resonance-state tracking method is used to compensate for frequency shifts caused by changes in temperature, pressure, and height, which affect the resonance frequency. In practice, the height of the installed ultrasonic resonance wind speed and direction sensor remains relatively constant, and the pressure in the working environment is also mostly stable. Thus, the scan range primarily focuses on temperature variation. Experiments have shown that a frequency scan range of 29 kHz to 42 kHz can meet the temperature requirements from −40 °C to 60 °C. The scan step is set to 100 Hz per scan, and after each scan, the corresponding resonance frequency is recalculated. The process is illustrated in Figure 9. Figure 9 shows the frequency scanning schematic and the resonance frequency point position schematic. In each cycle, different temperatures, pressures, and heights lead to variations in the resonance frequency point. The resonance point is fa in the first cycle, fb in the second, and fc in the third. In different cycles, the resonance point frequency shifts. Since pressure and height typically do not change drastically in normal measurement environments, the frequency shift of the resonance point is mainly related to temperature [18,19,20,21].

**Figure 10:**
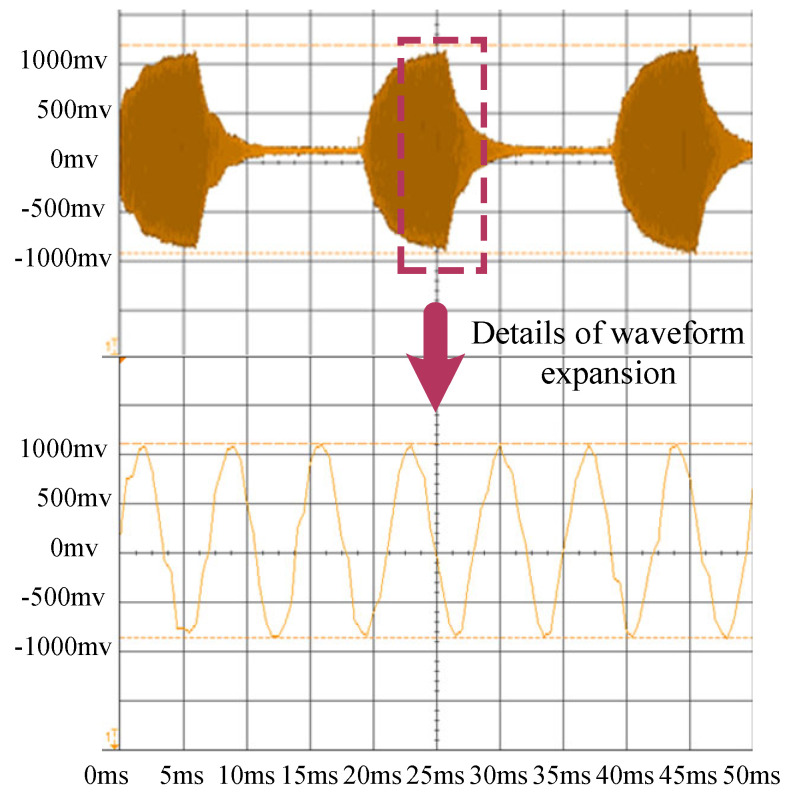
Resonance frequency test of ultrasonic resonance wind Sensor 1.

**Figure 11:**
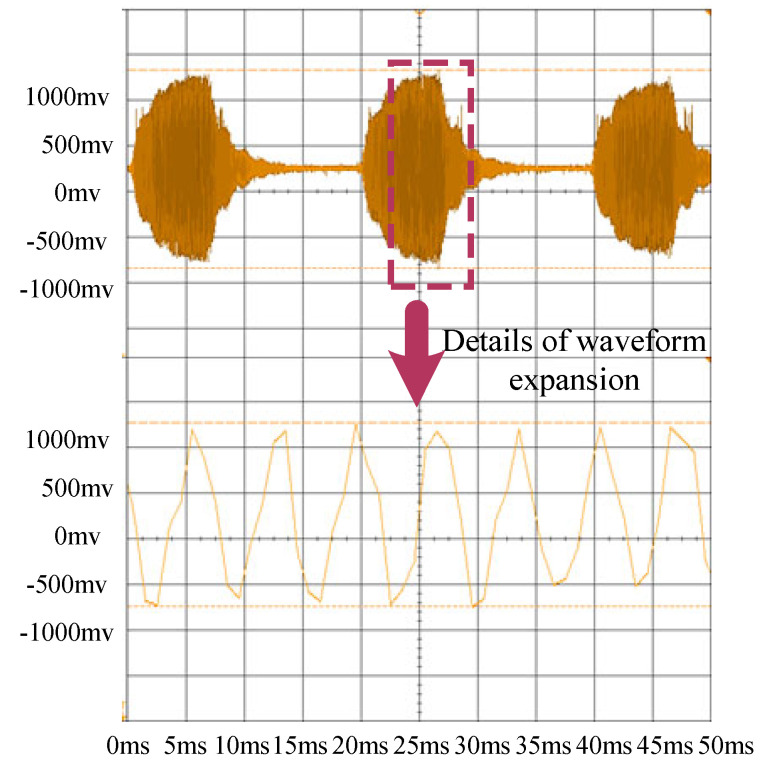
Resonance frequency test of ultrasonic resonance wind Sensor 2.

Based on the adaptive resonance-state tracking model, resonance frequency tests were conducted for ultrasonic resonance wind speed and direction Sensor 1 and Sensor 2 at the same location under normal temperature conditions. The test results are shown in Figure 10 and Figure 11. Figure 10 shows the overall waveform of Sensor 1 and the detailed waveform of Sensor 1. Figure 11 shows the overall waveform of Sensor 2 and the detailed waveform of Sensor 2.

As shown in Figure 10 and Figure 11, the resonance frequency of Sensor 1 is 36.4 kHz, while that of Sensor 2 is 36.8 kHz, with a frequency difference of 400 Hz. However, both sensors produce typical resonance waveforms. The adaptive resonance-state tracking method effectively achieves resonance frequency tracking. The frequency difference between the two sensors is due to machining errors during the manufacturing process and assembly errors, leading to inconsistent cavity heights in different sensors. These accumulated errors cause different resonance frequency points under the same conditions. Based on this characteristic, initial frequency calibration was performed in the actual compensation method.

### 4.3. Implementation of Resonance-State Temperature Compensation

As discussed above, the resonance frequency of the ultrasonic resonance wind speed and direction sensor varies primarily with temperature. Based on this principle, the current external environmental temperature can be inferred from the resonance frequency, eliminating the need for a temperature sensor. The temperature inferred from the resonance frequency is used as the temperature value for calculating the speed of sound, allowing for the accurate calculation of the ultrasonic wave speed during actual measurements, which can then be used for wind speed and direction compensation [21,22,23,24].

Ultrasonic resonance wind speed and direction Sensors 1 and 2 were tested at different temperatures in an mc711 temperature chamber, which has a precision of 0.1 °C and a resolution of 0.01 °C. Tests were conducted at temperature points ranging from −50.00 °C to 60.00 °C, with measurements taken every 10 °C. Due to the precision of the temperature chamber, the exact temperature points could not always be reached. The resonance frequencies at different temperatures were recorded, as shown in Table 3.

**Figure 12:**
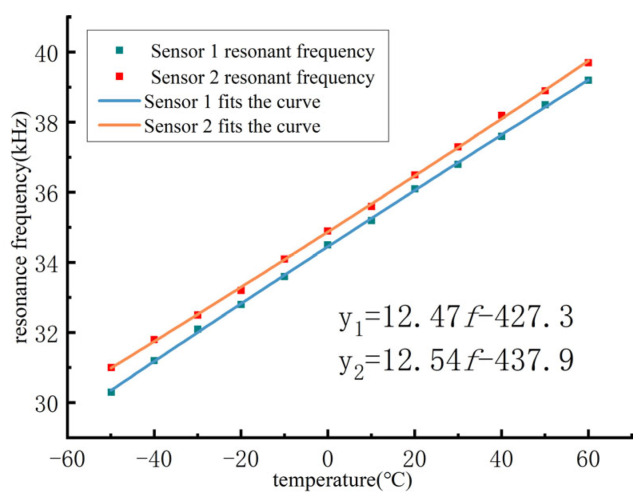
Resonance frequency variation with temperature.

Based on Table 3, the corresponding curves were plotted, showing the relationship between the resonance frequency and temperature, as illustrated in Figure 12.

As shown in Figure 12, the curve representing the resonance frequency variation with temperature for Sensor 1 was fitted to a linear relationship, with the equation expressed as follows:

(21) T=12.47×f−427.3,

Similarly, for Sensor 2, the curve was also fitted to a linear relationship, expressed as follows:

(22) T=12.54×f−437.9,

From these fitted equations, it is evident that both curves have the same slope, with the difference being a vertical shift along the *y*-axis. This difference can be attributed to accumulated machining and assembly errors, leading to different resonance frequency points for different ultrasonic resonance wind speed and direction sensors at the same temperature.

To verify the distribution of resonance frequencies under identical conditions and the consistency of frequency change rates at different temperatures, 10 different ultrasonic resonance wind speed and direction sensors were tested at 25 °C and 40 °C. These sensors had identical machining heights, and no variations in cavity machining height were used in this test, as cumulative machining and assembly errors after final production result in actual cavity heights differing from the design height. Testing multiple sensors with the same machining height yields results more representative of real-world applications. The 25 °C and 40 °C conditions were provided by the temperature chamber, and the resonance frequencies of the different sensors are shown in Table 4.

**Figure 13:**
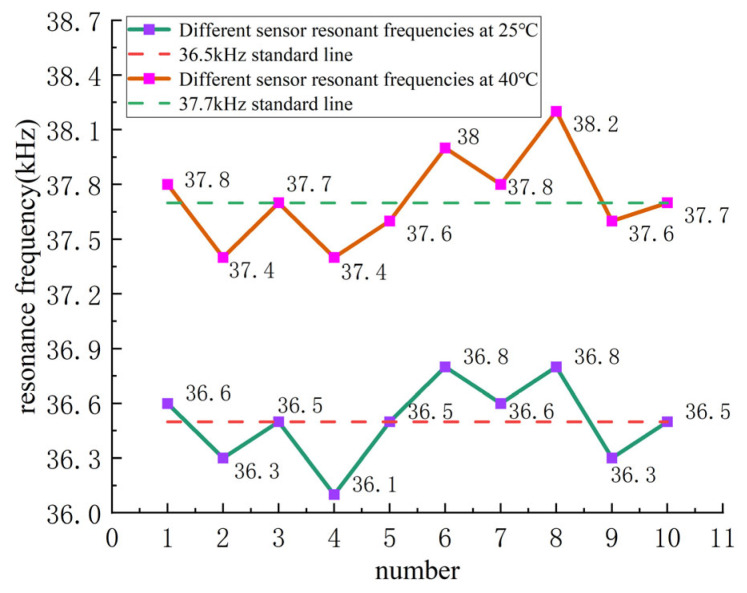
Comparison of resonance frequencies of different ultrasonic resonance wind sensors at 25 °C and 40 °C.

From Table 4, the corresponding curves were plotted, as shown in Figure 13.

**Figure 14:**
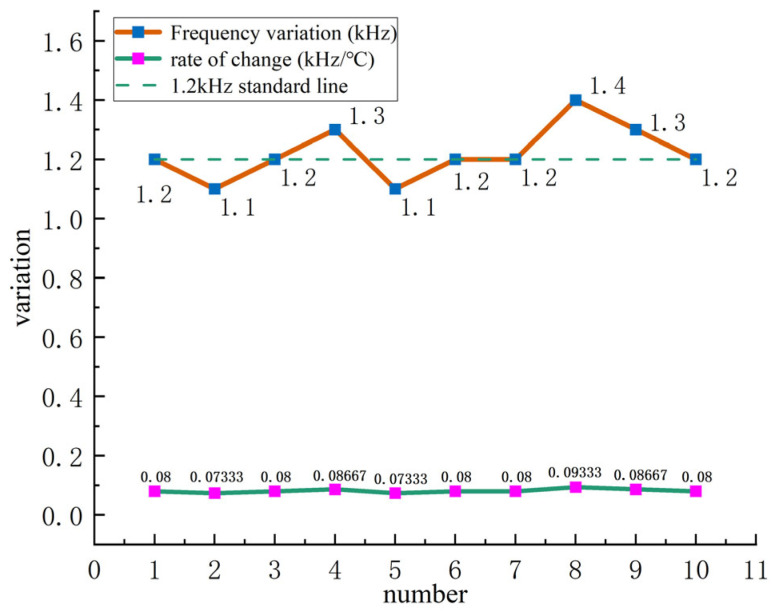
Frequency deviation and temperature change rate of different ultrasonic resonance wind sensors at 25 °C and 40 °C.

As shown in Figure 13, the resonance frequencies of the sensors at 25 °C are distributed between 36.1 kHz and 36.8 kHz, with a central line at 36.5 kHz. At 40 °C, the resonance frequencies are distributed between 37.4 kHz and 38.2 kHz, with a central line at 37.7 kHz. This demonstrates that, despite differences in sensor numbers, the deviation values and trends are generally consistent at different temperatures. The primary consideration for resonance-state temperature compensation is the relationship between the temperature and resonance frequency of the ultrasonic resonance wind speed and direction sensor. The frequency difference and change rate curves at different temperatures are shown in Figure 14.

As shown in Figure 14, the frequency deviation ranges from 1.1 kHz to 1.4 kHz, with an average value of 1.2 kHz. The rate of change ranges from 0.073 kHz/°C to 0.093 kHz/°C, with an average value of 0.08 kHz/°C. This indicates that for every 1 °C change in temperature, the corresponding frequency changes by approximately 0.08 kHz. The fact that the change rates of different ultrasonic resonance wind speed and direction sensors are nearly identical suggests that using resonance frequency for temperature compensation is feasible. The basic compensation equation can be expressed as follows:

(23) $$f_{\mathrm{resonant}\;\mathrm{frequency}}=f_{\mathrm{original}\;\mathrm{frequency}}+0.08*T,$$

Additionally, based on the test results shown in Figure 10 and Figure 11, each ultrasonic resonance wind speed and direction sensor has unique height errors due to machining and assembly. These height differences result in varying resonance frequencies. Therefore, the initial resonance frequency must be determined. By testing the resonance frequency at a specific temperature *T* and recording the actual working temperature, the initial frequency value can be calculated. The relationship between the working temperature and frequency is expressed as follows:

(24) $$T=12.5*f_{\mathrm{resonant}\;\mathrm{frequency}}-12.5*f_{\mathrm{original}\;\mathrm{frequency}},$$

Where $f_{\mathrm{original}\;\mathrm{frequency}}$ is a known value, and the relationship between temperature and resonance frequency is linear.

From Equations (23) and (24), the compensated speed of sound can be calculated as follows:

(25) $$c=331.45+0.606\times \left(12.5*f_{\mathrm{resonant}\;\mathrm{frequency}}-12.5*f_{\mathrm{original}\;\mathrm{frequency}}\right)$$

Equation (25) establishes a linear relationship between the resonance frequency *f* and the speed of sound *c*. Using this equation, the actual working temperature of the ultrasonic resonance wind speed and direction sensor can be determined based on the resonance frequency points obtained during frequency scanning. The actual speed of sound c can then be calculated without the use of a temperature sensor, effectively achieving temperature compensation based on the resonance state.

## 5. Experimental Test Results

**Figure 15:**
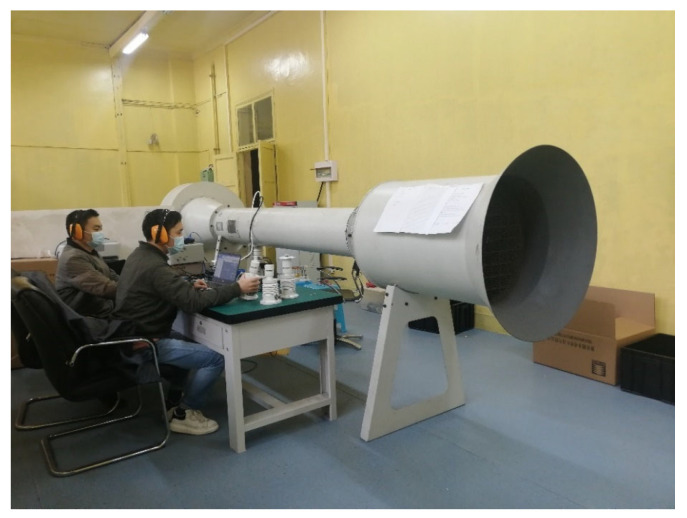
Wind tunnel calibration test system.

The ultrasonic resonance wind speed and direction sensor with resonance-state temperature compensation was tested. The testing system, as shown in Figure 15, includes a high-precision wind tunnel, a high-precision encoder, a wind speed standard source, calibration testing software, and a specialized fixture [25]. The wind tunnel used is a custom RE-2000 DC wind tunnel, with a wind speed output range of 0–60 m/s. The high-precision encoder is model AR60-40, with a rotation angle range of 0°–360° and a rotational accuracy of 0.02°. The wind speed standard source is a calibrated CEM high-precision differential pressure gauge DT8920, with a wind speed measurement range of 1.00–80.00 m/s and a resolution of 0.001 m/s, calibrated to an accuracy of 0.01 m/s.

Both the ultrasonic resonance wind speed and direction sensor with resonance-state temperature compensation and the sensor without temperature compensation were tested under the same conditions in the wind tunnel, at morning and evening temperatures of 17 °C and 29 °C, respectively. Due to the large size of the wind tunnel, experiments could not be conducted inside a temperature chamber, so natural temperature variations were used for validation. Wind speeds of 0.00 m/s, 3.50 m/s, 10.00 m/s, 20.00 m/s, 34.00 m/s, and 50.00 m/s were tested. As the wind tunnel could not precisely reach the target values, fine control was used to approximate the required values. The wind speed standard source was the calibrated CEM high-precision differential pressure gauge DT8920. The test data are shown in Table 5 and Table 6.

**Figure 16:**
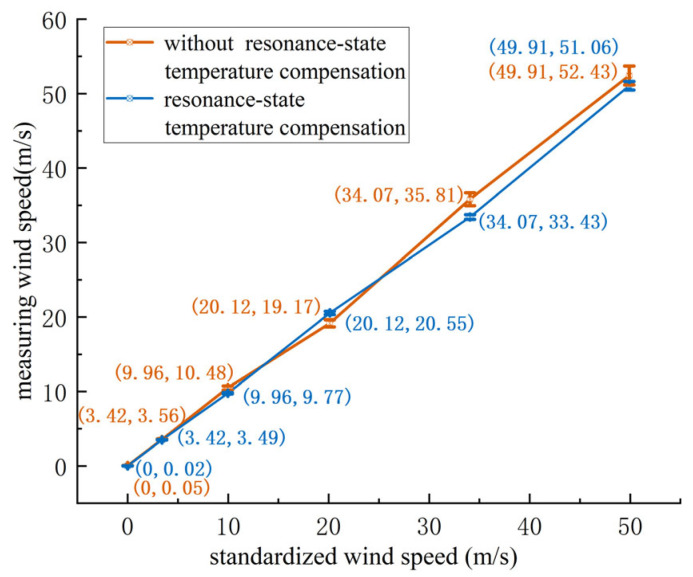
Wind speed measurement and standard deviation at 17 °C.

Based on Table 5, the corresponding curve was plotted, showing the wind speed measurements and standard deviation at 17 °C, as depicted in Figure 16.

As shown in Figure 16, at 17 °C, as the wind speed increases, the standard deviation for both the compensated and non-compensated sensors increases. However, the maximum deviation for the sensor with resonance-state temperature compensation is 2.3%, compared to 5.2% for the non-compensated sensor. The sensor with resonance-state temperature compensation outperformed the non-compensated sensor at every test point.

**Figure 17:**
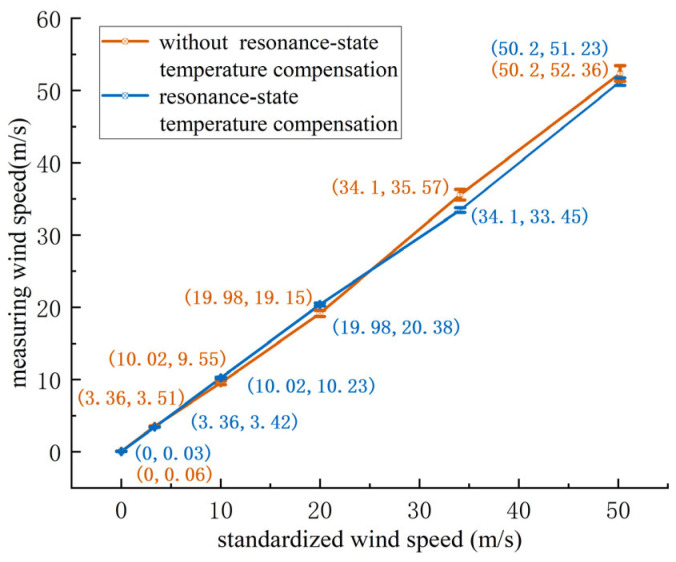
Wind speed measurement and standard deviation at 29 °C.

Based on Table 6, the corresponding curve was plotted, showing the wind speed measurements and standard deviation at 29 °C, as depicted in Figure 17.

As shown in Figure 17, at 29 °C, as the wind speed increases, the standard deviation for both the compensated and non-compensated sensors increases. The maximum deviation for the sensor with resonance-state temperature compensation is 2.1%, compared to 4.6% for the non-compensated sensor. Again, the sensor with resonance-state temperature compensation outperformed the non-compensated sensor at each test point.

In summary, the non-compensated ultrasonic resonance wind speed and direction sensor showed a 5.2% error at 17 °C and a 4.6% error at 29 °C. This is because the internal temperature of the sensor circuit board is around 40 °C, and the closer the external temperature is to this value, the smaller the wind speed measurement error. The compensated sensor exhibited errors of 2.3% at 17 °C and 2.1% at 29 °C, with measurement accuracy remaining consistent across different temperatures. The experiments demonstrate that resonance-state temperature compensation effectively improves the accuracy of wind speed and direction measurements. The wind speed measurement accuracy is ±0.3 m/s (≤15 m/s)/±2.3% (15 m/s–50 m/s), which is superior to the traditional ultrasonic wind speed and direction sensors’ accuracy of ±0.5 m/s (≤15 m/s)/±4% (15 m/s–50 m/s). Compared to the non-compensated sensor, the accuracy improved by approximately 3%; the consistency of wind speed measurement is ≤±0.3%.

## 6. Conclusions

The findings of this study are as follows:

1. This method represents a novel approach to temperature compensation for ultrasonic wind speed measurement. It achieves temperature compensation through resonance frequency without the use of temperature sensors. This approach addresses the issue of temperature compensation errors arising from inconsistencies between internal and external temperatures during the operation of ultrasonic resonance wind speed and direction sensors. It provides effective compensation, particularly in various environments, including cold and icy conditions where heating is required for the ultrasonic wind sensor to function properly. The proposed resonance-state temperature compensation method solves the problem of temperature compensation errors caused by inconsistent internal and external temperatures during the operation of the ultrasonic resonance wind speed and direction sensor. The compensated sensor improved the measurement accuracy by approximately 3% compared to the non-compensated sensor, with deviations of ≤±0.3% across different temperatures. The overall wind speed measurement accuracy was superior to traditional ultrasonic wind speed and direction sensors. Resonance-state temperature compensation effectively enhances the accuracy and stability of wind speed and direction measurements.
2. The resonance-state temperature compensation method is widely applicable to ultrasonic resonance wind speed and direction sensors. Given a fixed resonance cavity height, the temperature-to-resonance frequency change rate is approximately 0.08 kHz/°C.
3. Due to cumulative machining and assembly errors, each ultrasonic resonance wind speed and direction sensor has different initial resonance frequencies caused by height variations. The initial frequency needs to be calculated and corrected to accurately apply resonance-state temperature compensation.

## Figures and Tables

**Table 1:** Comparison of temperature compensation methods.

Method	Advantages	Disadvantages	Applicable Scenarios
Time-Of-Flight Estimation Method.	No additional components required.	In heating conditions, the differing temperatures at the solid–gas–solid interface of the ultrasonic transducer can lead to inaccuracies in the receiving time, resulting in significant variations in temperature compensation across different temperatures.	Commonly used for indoor temperature measurements; not suitable for extreme environments.
Temperature Sensor Measurement Method.	Direct temperature measurement ensures high reliability.	Requires additional components; in heating conditions, the temperature sensor is located inside the wind sensor, and the temperature difference between the interior and exterior can cause substantial deviations in temperature compensation.	Typically used for outdoor temperature compensation without heating; not suitable for extreme environments.
Resonance-State Temperature Compensation.	No additional components; strong correlation with temperature allows for good compensation linearity and high accuracy.	Requires initial calibration of resonance frequency.	Applicable in various environmental conditions.

**Table 2:** Ultrasonic air propagation speed at different temperatures and pressures.

Temperature (°C)	Pressure1 (1 Bar)	Pressure2 (6 Bar)	Pressure3 (12 Bar)
−8	324.69	322.09	319.06
0	330.67	328.35	325.68
10	337.95	335.95	333.66
20	345.01	343.29	341.34
50	365.09	364.03	362.88

**Table 3:** Resonance frequency testing at different temperatures.

Temperature (°C)	Resonance Frequency of Sensor 1 (kHz)	Resonance Frequency of Sensor 2 (kHz)
−49.92	30.3	31.0
−40.03	31.2	31.8
−29.97	32.1	32.5
−20.01	32.8	33.2
−10.02	33.6	34.1
−0.06	34.5	34.9
10.03	35.2	35.6
20.05	36.1	36.5
29.99	36.8	37.3
40.02	37.6	38.2
50.05	38.5	38.9
60.01	39.2	39.7

**Table 4:** Resonance frequencies of different ultrasonic resonance wind speed and direction sensors at 25 °C and 40 °C.

Sensor Number	Resonance Frequency at 25 °C (kHz)	Resonance Frequency at 40 °C (kHz)
1	36.6	37.8
2	36.3	37.4
3	36.5	37.7
4	36.1	37.4
5	36.5	37.6
6	36.8	38.0
7	36.6	37.8
8	36.8	38.2
9	36.3	37.6
10	36.5	37.7

**Table 5:** Wind speed test results at 17 °C.

Wind Speed (m/s)	Without Compensation (m/s)	With Resonance-State Temperature Compensation (m/s)
0.00	0.05	0.02
3.42	3.56	3.49
9.96	10.48	9.77
20.12	19.17	20.55
34.07	35.81	33.43
49.91	52.43	51.06

**Table 6:** Wind speed test results at 29 °C.

Wind Speed (m/s)	Without Compensation (m/s)	With Resonance-State Temperature Compensation (m/s)
0.00	0.06	0.03
3.36	3.51	3.42
10.02	9.55	10.23
19.98	19.15	20.38
34.10	35.57	33.45
50.20	52.36	51.23

## Data Availability

Data are contained within the article.
